# Transcriptome Analysis of Ovarian Follicles Reveals Potential Pivotal Genes Associated With Increased and Decreased Rates of Chicken Egg Production

**DOI:** 10.3389/fgene.2021.622751

**Published:** 2021-03-10

**Authors:** Xiaoxia Chen, Xue Sun, Ignatius Musenge Chimbaka, Ning Qin, Xiaoxing Xu, Simushi Liswaniso, Rifu Xu, John Michael Gonzalez

**Affiliations:** ^1^Department of Animal Genetics, Breeding and Reproduction, College of Animal Science and Technology, Jilin Agricultural University, Changchun, China; ^2^Joint Laboratory of Modern Agricultural Technology International Cooperation, Ministry of Education, Jilin Agricultural University, Changchun, China; ^3^College of Agricultural & Environmental Sciences, University of Georgia, Athens, GA, United States

**Keywords:** chicken, egg production, differentially expressed gene, ovarian follicle, transcriptome

## Abstract

Egg production is an important economic trait in the commercial poultry industry. Ovarian follicle development plays a pivotal role in regulation of laying hen performance and reproductive physiology. However, the key genes and signaling pathways involved in the various-stages of laying hen follicular development remain poorly understood. In this study, transcriptomes of ovarian follicles at three developmental stages, the large white follicle (LWF), small yellow follicle (SYF), and large yellow follicle (LYF), were comparatively analyzed in hens with high (HR) and low (LR) egg-laying rates by RNA-sequencing. Eighteen cDNA libraries were constructed and a total of 236, 544, and 386 unigenes were significantly differentially expressed in the LWF, SYF, and LYF follicles of HR and LR hens, respectively. Among them, 47 co-transcribed differentially expressed genes (DEGs) in LWF and SYF, 68 co-expressed DEGs in SYF and LYF, and 54 co-expressed DEGs in LWF and LYF were mined. Thirteen co-expressed DEGs were found in LWF, SYF, and LYF follicles. Eighteen candidate genes, including *P2RX1*, *CAB39L*, *BLK*, *CSMD3*, *GPR65*, *ADRB2*, *CSMD1*, *PLPP4*, *ATF3*, *PRLL*, *STMN3*, *RORB*, *PIK3R1*, *PERP1*, *ACSBG1*, *MRTO4*, *CDKN1A*, and *EDA2R* were identified to be potentially related to egg production. Furthermore, Kyoto Encyclopedia of Genes and Genomes analysis indicated neuroactive ligand-receptor interaction, cell adhesion molecules, peroxisome proliferator-activated receptor pathway, and cAMP signaling pathway might elicit an important role in formation of egg-laying traits by influencing ovarian follicle development. This study represents the first transcriptome analysis of various-sized follicles between HR and LR hens. These results provide useful molecular evidence for elucidating the genetic mechanism underlying ovarian follicle development associated with egg production in chicken.

## Introduction

Egg-laying production efficiency is an important economic trait in the world-wide poultry industry. Efficiency is determined by the number of ovarian follicles destined for ovulation or atresia and the capacity of the oviduct to transform the ova into a hard-shelled egg (Etches et al., 1990; Luan et al., 2014). Follicle development is an intricate and highly coordinated cellular process, which is characterized by a well-organized follicular hierarchy in high production egg-laying hens. In chickens with low egg-laying rates, such as the broiler breeder hen, follicular development is not well-organized, which results in reduced reproductivity (Johnson, 2012). Growing follicles are mainly classified into primordial follicles (smaller than 0.08 mm in diameter), primary follicles (from 0.08 mm up to 1 mm in diameter), undifferentiated prehierarchical follicles (from 1 mm up to 8 mm in diameter), and preovulatory/hierarchical (larger than 9 mm in diameter) follicles (Lovell et al., 2003; Onagbesan et al., 2009). During growth and development of the hen ovary, a majority of the follicles undergo atresia, resulting in a limited number of follicles further developing into preovulatory follicles. In this process, follicle selection (cyclic recruitment) plays a critical role in determining whether prehierarchical follicles enter the follicular hierarchy or undergo atresia in the ovary. During follicle selection, only one dominant follicle is generally recruited into the preovulatory hierarchy on an approximate daily basis, which occurs from a small cohort of prehierarchical follicles measuring approximately 6.0- to 8.0-mm in diameter (Woods and Johnson, 2005; Johnson, 2012). In different ovarian follicle developmental stages, many divergent biological processes affect oocyte growth, and proliferation and differentiation of granulosa and theca cells within the various-sized follicles. Moreover, at the different follicular stages, studies reported ovaries were strictly controlled by multiple distinct reproductive endocrine hormones, paracrine and autocrine regulatory factors, as well as regulatory activities of hypothalamic-pituitary-gonadal axis (Padmanabhan et al., 2002; Johnson and Woods, 2009). Previous studies reported various follicular sizes exhibited different effects on granulosa cell apoptosis, autophagy, and follicular atresia (Lin and Rui, 2010; Zheng et al., 2019). Furthermore, numerous studies established ovarian follicular function is regulated by a plethora of endocrine, paracrine, and autocrine factors besides the pituitary gonadotropins (follicle stimulating hormone and luteinizing hormone) including steroids, neuropeptides, growth factors, adipokines, cytokines, transcription factors, and so on (Woods and Johnson, 2005; Onagbesan et al., 2009; Dupont et al., 2012; Johnson, 2015b; Xu et al., 2018). Obviously, follicular size has a close relationship with its developmental biology, physiological function, and molecular regulation.

In recent years, transcriptome analyses revealed and identified many genes relevant to high egg production and which are expressed specifically in ovaries and the glands of the hypothalamus and pituitary of chickens. There are five differentially expressed genes (DEGs) that were identified in the ovaries of Jinghai Yellow chickens related to egg production including *ZP2*, *WNT4*, *AMH*, *IGF1*, and *CYP17A1*, which serve as potential candidate genes for improving egg production (Zhang et al., 2019). It was reported that 414, 356, and 10 DEGs from the hypothalamus, pituitary gland, and ovary, respectively, of the Chinese domestic Luhua chicken were potentially associated with greater rates of egg production (Mishra et al., 2020). These results provided valuable information for understanding the reproductive biology of chicken and identifying effective molecular markers for genetic improvement of the domestic chicken breed. Another report has demonstrated high- and low-yielding Chinese Dagu hens possessed 7 and 39 DEGs in the hypothalamus and pituitary, respectively. The main DEGs identified by the authors consisted of *OVCH2*, *SLC7A10*, *VCAN*, *CATHL2*, *GFRA4*, *ESR1*, *LAMA1*, *CFD*, and *MANBAL1* (Wang and Ma, 2019). Additionally, an earlier study identified transcripts in the hypothalamus and pituitary gland of Taiwan Country chickens including *PLAG1*, *NCAM1*, *PRL*, *SAR1A*, *BDH*, *PCDHA@*, *PGDS*, *SCG2*, and *STMN2*, which could serve as potential molecular markers of high egg production in chicken breeding (Shiue et al., 2006).

Several studies in the literature reported similar findings in geese and ducks. One study identified 26 genes (18 known and 8 unknown DEGs) in the ovaries of pre-laying and laying geese, with *FSHR*, *ESR1*, *ESR2* and *PRLR*, and *FTH* serving as representative candidate genes (Kang et al., 2009). Luan et al. (2014) identified 12 candidate genes involved in reproduction regulation that differed during the laying and termination period of egg production of the Huoyan goose breed. This study provided transcriptome profiling data that increased our understanding of geese reproductive biology. Tao et al. (2017) verified 30 genes were relevant to the reproductive process, with 25 associated with egg production in the ovaries of laying ducks. These findings provided useful information that may contribute to future functional studies of genes involved in bird reproduction (Tao et al., 2017). Ren et al. (2019) found seven key DEGs, including *EPOX*, *StAR*, *CYP17*, *3β-HSD*, *CYP1B1*, *CYP19A1*, and *SR-B1*, were related to Peking duck ovarian follicle development (Ren et al., 2019). Wu et al. (2020) revealed 135, 56, and 331 genes in Xinjiang Yili geese hypothalamus, pituitary gland, and ovaries, respectively, were related to high and low egg production (Wu et al., 2020). These results provided transcriptome data of the Xinjiang Yili goose hypothalamic-pituitary-gonadal axis, which laid a foundation for further exploration of the molecular mechanisms underlying egg-laying performance of birds. However, the role and molecular regulation DEG play in the formation of various-sized ovarian follicles and egg-laying traits are still poorly understood.

The Lohmann Brown (LB) layer, a commercial egg-laying breed, is characterized as possessing a high rate of egg production (HR), high laying peak, and great consistency in laying performance (Lohmann Tierzucht GmBH, 2005). In contrast, the Jilin Black (JB) chicken, an indigenous Chinese breed (dual-purpose type for egg and meat production), is characterized by a low rate of egg production (LR), low laying peak, and strong broodiness (Chen et al., 2004). The divergences in the egg-laying performance between LB and JB, therefore, may serve as a proper model for discovering potential key genes involved in egg production and for further investigating the molecular mechanism underlying formation of egg-laying traits in chicken.

To reveal the potential candidate genes associated with egg production, high-throughput transcriptome technology was performed to enrich and analyze differentially expressed genes of follicles sized 3.5–5.5, 6.0–8.0, and 8.5–10.5 mm in diameter between the LB and JB breeds. The objective of this study was to identify key functional genes implicated in ovarian follicular growth and development (including follicle selection) that directly influences egg-laying productivity in hens, and to explore molecular functions, biological processes, and signaling pathways by which the candidate genes contribute to egg-laying production. The present study has laid a foundation for further work ascertaining the exact roles and regulatory mechanisms of the promising genes in ovary development. Finally, these results can be utilized for discovering potential molecular markers of egg-laying traits that can facilitate layer breeding and genetic improvement programs.

## Materials and Methods

### Ethics Statement

All procedures executed in chickens were approved by the Institutional Animal Care and Use Committee (IACUC) of Jilin Agricultural University (Changchun, China). All birds were sacrificed prior to the removal of organs based upon the IACUC Guidelines for the euthanasia of experimental animals [Permission No. GR (J) 19-030]. Euthanasia of the chickens is conducted fully compliant with Chinese applicable laws and regulations concerning care and use of laboratory animals, which was issued on the basis of the Regulations for the Administration of Affairs Concerning Experimental Animals of the State Council of the People’s Republic of China (1988). All of efforts have been made to minimize the suffering of the animals.

### Animals and Samples Preparation

Lohmann Brown and Jilin Black hens were reared in laying battery cadges according to the husbandry practices as previously reported (Qin et al., 2015). Under the feeding conditions, average body weights of LB and JB hens at the age of 21 weeks were 1.71 ± 0.13 kg and 1.78 ± 0.19 kg, average egg production rate at the age were 53 and 11%, respectively. Ten birds of each breed were obtained from the population, euthanized at 21 weeks of age, and their ovarian follicles were harvested. Growing follicles were categorized by size (3–5 or 6–8 mm) or according to color (large white follicles or small yellow follicles) types (Johnson and Woods, 2009; Johnson, 2012). In this study, white follicles 3.5–5.5 mm in diameter were categorized as large white follicles (LWF), yellow follicles 6.0–8.0 mm were categorized as small yellow follicles (SYF), and yellow follicles 8.5–10.5 mm were categorized as large yellow follicles (LYF, Supplementary Figure 1). After the surrounding vascular and connective tissues of the follicles were removed with fine forceps and a scalpel (Politis et al., 1990), follicles were immediately snapped frozen in liquid nitrogen and preserved at –80°C for RNA extraction.

Total RNA was isolated from follicles of each hen using Trizol Reagent (Invitrogen Life Technologies, Carlsbad, CA, United States) according to the recommended manufacturer’s protocol, and concentration, quality, and integrity were determined by a Thermo NanoDrop 8000 Spectrophotometer (Thermo Fisher Scientific, Wilmington, DE, United States). Three micrograms of RNA were used as input material for RNA sample preparations. The other parts of the samples were stored at –80°C for validation experiments.

### cDNA Library Construction and Illumina Sequencing

cDNA libraries were generated using the TruSeq RNA Sample Preparation Kit (Illumina, San Diego, CA, United States). Briefly, mRNA was purified from total RNA using poly-T oligo-attached magnetic beads. First strand cDNA was synthesized using random oligonucleotides and SuperScript II after fragmentation was performed utilizing divalent cations under elevated temperature in an Illumina proprietary fragmentation buffer. Second strand cDNA synthesis was subsequently carried out by using DNA polymerase I and RNase H. Remaining overhangs were converted into blunt ends via exonuclease/polymerase activities and enzymes were removed. Following adenylation of the DNA fragment 3′ends, Illumina PE adapter oligonucleotides were immediately ligated to prepare for hybridization. To screen cDNA fragments 200 bp in length, library fragments were purified by the AMPure XP system (Beckman Coulter, Beverly, CA, United States). DNA fragments with ligated adaptor molecules on both ends were selectively enriched by applying Illumina PCR Primer Cocktail in a 15 cycle PCR reaction. Finally, products were purified (AMPure XP system) and quantified utilizing the Agilent high sensitivity DNA assay on a Bioanalyzer 2100 system (Agilent Biotechnologies, Palo Alto, CA, United States).

Effective concentration of libraries were greater than 2 nM and libraries were qualified, sequenced, and paired-end sequencing with the 150 bp sequencing read length was performed. The cDNA libraries were sequenced on a Hiseq platform (Illumina, Inc., San Diego, CA, United States) by Shanghai Personal Biotechnology Cp. Ltd.

### RNA-Seq Data Analysis

To obtain clean reads (clean data), raw data (raw reads) of FASTQ format^1^ were processed by filtering out low-quality reads and trimming the adaptor sequences using in-house perl scripts. Meanwhile, Q20, Q30, and N (fuzzy base) content of the raw data were evaluated. Follow-up analyses were based on clean data with high quality. All of the clean reads were aligned with the reference genome^2^ by using sequence alignment program HISAT 2.1.0 (Pertea et al., 2016; Kim et al., 2019).

### Identification of Differentially Expressed Genes

HTSeq (ver. 0.6.1) software was used to count the reads mapped to each gene. The gene expression level was normalized based on its FPKM (Fragments per Kilobase of transcript per Million mapped reads) values using the Cufflinks package (v2.1.1). The expression pattern assessment for differentially expressed genes (DEGs) was performed by the Multi-Experiment Viewer (MeV) software (version 4.9.0)^3^ based upon the normalized FPKM + 1 value between case group and control group. The resulting *p*-values were adjusted using the Benjamini and Hochberg’s approach for controlling the false discovery rate and an absolute value of the |log2FoldChange| (Log2FC) was used as the threshold for judging significance of the gene expression. Candidate genes with an adjusted *p*-value (padj) < 0.05 and Log2FC > 1 were identified as differentially expressed. Volcano plots to visualize up- and down-regulated genes of each sample were generated by using the R package “ggplot2” (version 3.5.0), based on the values of padj and Log2FC (Wang et al., 2010). To explore the similarity of these samples based on their mRNA expression pattern, all statistical analyses were performed in the R statistical program, and Principle component analysis (PCA) was finished using companion to applied regression (CAR) package in R^4^.

### GO and KEGG Enrichment Analysis for DEGs

An online biological tool, Gene Ontology (GO^5^), was utilized to annotate and analyze the molecular and functional characteristics of the DEGs (Ashburner et al., 2000). The calculated *p*-value was Bonferroni corrected, taking the corrected *p*-value < 0.05 as a threshold for GO annotation, by which the DEGs were enriched in three GO terms including biological process (BP), cellular component (CC), and molecular function (MF) of the GO database. A topGO version 3.0 package^6^ provided the tools for testing GO terms while accounting for the topology of the GO graph.

Another online biological tool, KEGG (Kyoto Encyclopedia of Genes and Genomes^7^), provided the comprehensive database resources for the KEGG pathway enrichment analysis of the DEGs. In this step, four databases were utilized to reveal high-level functions and biological systems of the DEGs, including Reactome^8^, KEGG pathway^9^, PANTHER^10^, and STRING^11^. Results with *p* < 0.05 were considered significantly enriched by DEG. Additionally, the UniProt (UniProt Knowledgebase^12^) database, EC (Enzyme Commission^13^) database, and eggNOG (Evolutionary Genealogy of Genes: Non-supervised Orthologous Groups^14^) database were used for functional and biological analysis of the enriched DEGs.

### Data Validation by Quantitative Real-Time RT-PCR

To verify the accuracy and repeatability of the RNA-Seq results of EDGs, transcription levels of 22 selected genes in the follicles were assessed by using quantitative real-time reverse transcriptase PCR (RT-qPCR) according to our previously described method (Xu et al., 2018). The primers utilized for amplification of the candidate genes including *P2RX1*, *CAB39L*, *BLK*, *CSMD3*, *GPR65*, and *ADRB2*, et al., were listed in Table 1. Using the 2^–ΔΔCt^ method, mRNA expression results were normalized against*18S rRNA* as internal control. To quantify mRNA expression levels by RT-qPCR analysis, four amplified products in independent reactions per bird were utilized. All the experiments were carried out in triplicate using different batches of sampled follicles.

**TABLE 1:** Primer pairs designed for quantitative real-time PCR analysis.

Gene	Forward primer (5′ – 3′)	Reverse primer (5′ – 3′)	Accession No.	Size
*P2RX1*	TTTCCTCTATGAGAAGGGCTAC	GGGCAGGTTCCTTGTTTC	NM_204519	210 bp
*CAB39L*	GATGTCCCAGTGATGTGC	AATTCAGCCTCTTGCTCT	NM_001006272	150 bp
*BLK*	GATGCTGGCGAAACAAAC	GGCTCTGCCTCATACTGC	XM_004935895	103 bp
*CSMD3*	AGGTGGACAACTCAACAG	GGTGCCAACTAAGTAATCT	XM_015283035	205 bp
*GPR65*	TGCCTCTGTGGATTGATT	TCTTGTGCGTAAGTGCTG	XM_421305	185 bp
*ADRB2*	CGCTGTGGACCGATACTTT	AGATGATGGAGGAGGCAAT	MK139003	239 bp
*CSMD1*	AGGAGGATTCTTTGTTCT	GATGGCATTTGTACTTGA	XM_015284920	137 bp
*PLPP4*	TTTGGATGGCAGATTGTC	CAGTCTAAAGCACCGTAA	XM_426544	172 bp
*ATF3*	GGTGACAGTGTCCGAAAG	TATCAAATGCTGCTTCTC	XM_015283874	238 bp
*PRLL*	GCAGTAGATGAAGCGATGT	GCCATACCCAACTCCCAC	NM_001165912	172 bp
*STMN3*	GAAGTCCGCAGGAACAAG	AACGCTGGCATTTAGAACA	NM_001004393	144 bp
*RORB*	GGTGCTGAGACGGGATTT	GGAGTAGGAGGCATTGTTC	NM_205093	215 bp
*PIK3R1*	TGCCATTTGTTCCTGTCG	GCCCTAAGCATTTATCCC	XM_424759	150 bp
*PERP1*	AGACCTTGCCCTATGTGC	GAAGTTGAACCGAAGTGTAT	NM_001277703	206 bp
*ACSBG1*	TTACATAAGGGAGCATTTC	TAACTCATTGCCACCACT	XM_413747	103 bp
*MRTO4*	TACACCGTCAGCCTGGAC	CTCTGCCATCTCGTAGCC	XM_025142548	201 bp
*CDKN1A*	GACCACGGAAGGGACTGA	AGACGGTGACGGACCACA	NM_204396	129 bp
*EDA2R*	CCCTGGTCATCATCGTGC	GCTTCCACTGGTCCTTCTGT	NM_001083360	241 bp
*GABRA1*	TGTGTTTTCTGCCCTCATC	ATCCTTCACCTTCTTTGGC	NM_204318	109 bp
*NDUFAB1*	AGGACGAGTTCGGCTTTG	TGACATCAGGGCCATCC	XM_004945263	157 bp
*NCAM2*	CGGCTACAAACAGAATAGGAA	ATGAATGAAACCTTGGCAGT	XM_425540	124 bp
*LOC424014*	TGAGGATGGCTCGGTTGA	AATGGTGTAGGTGCTGATGG	XM_001232693	130 bp
*18S rRNA*	ATTGGAGGGCAAGTCTGGTG	CCAGCTCGATCCCAAGATCC	AF173612	109 bp

### Statistical Analysis

Statistical analysis was fulfilled using the SPSS12.0 software package (Lin et al., 2002). Data were analyzed by executing a Student’s *t-*test for comparisons between the RNA-Sequencing and RT-qPCR determination after confirmation of normal distributions for non-parametric analysis. Values were presented as mean ± SEM and bars with superscript symbols that indicate the significant difference compared with control groups at ^∗∗^*p* < 0.01, ^∗^*p* < 0.05.

## Results

### Overview of RNA Sequencing Data

**FIGURE 1:**
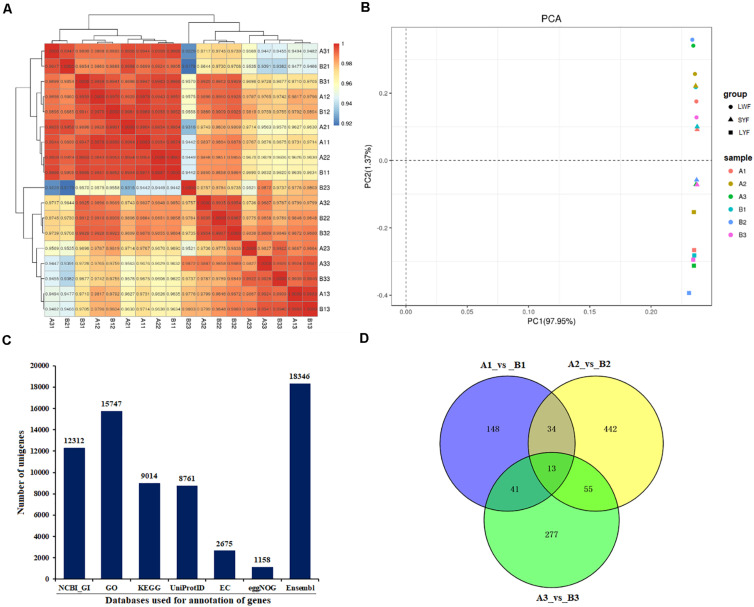
Basic information of sequencing data. **(A)** Pairwise Pearson’s correlation coefficients of sequencing data from six samples with three replicates; **(B)** Principal component analysis (PCA) of transcriptomic variation. Shapes denote different groups of the follicles sampled, colored dots indicate different samples; **(C)** annotation of gene numbers based on different databases; **(D)** Venn diagram analysis of distribution of annotated genes of different follicle groups of HR and LR hens. A1, LWF of HR hens; B1, LWF of LR hens; A2, SYF of HR hens; B2, SYF of LR hens; A3, LYF of HR hens; B3, LYF of LR hens.

In this study, a total of 18 cDNA libraries were constructed from HR and LR ovarian follicles ranging in size from 3.5 to 5.5 mm (LWF), 6.0 to 8.0 mm (SYF), and 8.5 to 10.5 mm (LYF) in diameter. Raw reads of each library ranged from 41.61 million up to 53.22 million reads. Among the raw reads, more than 97.35 and 93.87% had quality scores at the Q20 and Q30 levels, respectively. The highest N (base) content of the raw data was 0.0071% (Supplementary Table 1). By quality filtering to strain off low-quality and adaptor sequences, more than 41 million clean reads of each library were procured. In the clean data, clean reads of high quality accounted for 98.91–99.27% of the sequences of the raw reads, and bases (bp) of the clean data accounted for 99.17–99.56% of the raw data (Supplementary Table 2). Total mapped sequences (reads) possessed 85.11–86.49% of the clean reads. In the mapped reads, multiple mapped reads accounted for 3.54–5.25% of the clean reads, and uniquely mapped reads (URs) accounted for 94.75–96.46% of the clean reads (Supplementary Table 3). Among the RNASeq mapped events, total reads mapped to genes (MGs) shared 81.69–83.29% of the URs and mapped to genomic interval regions (interGene) shared 16.71–18.31%. Sequences mapped to exons covered 87.21–91.37% of the MGs (Supplementary Table 4). To quantify gene expression levels, Pairwise Pearson’s correlation coefficients of the samples with their biological replicates were evaluated higher than 0.9179, indicating high repeatability and reliability of the sequencing data (Figure 1A). To obtain an overview of the transcriptomic variation, principal component analysis (PCA) was performed and the values of PC1 and PC2 were 97.95 and 1.37%, respectively (Figure 1B). The genes that led to the maximum amount of variance (PC1) were selected and GO terms obtained by using the GO Consortium.

### Functional Annotation of Unigenes

After sequences of unigenes were aligned to the reference genome^15^ of the Ensembl database, unigenes were analyzed and annotated by using the NCBI and five other databases (Figure 1C). Total, 18,346 unigenes were annotated in the 18 cDNA libraries. Among them, there were 12,312 unigenes matched successfully in the NCBI databases, accounting for 67.10% of the total unigenes. The current data demonstrated that 15,747 unigenes (85.83%) had successfully matched in the GO database, 9,014 unigenes (49.13%) in KEGG database, 8,761 unigenes (47.75%) in UniProt ID database, and 2,675 unigenes (14.58%) in EC database, and 11,58 unigenes (6.31%) in egg NOG database (Figure 1C).

### Identification and Analysis of Differentially Expressed Genes (DGEs)

**FIGURE 2:**
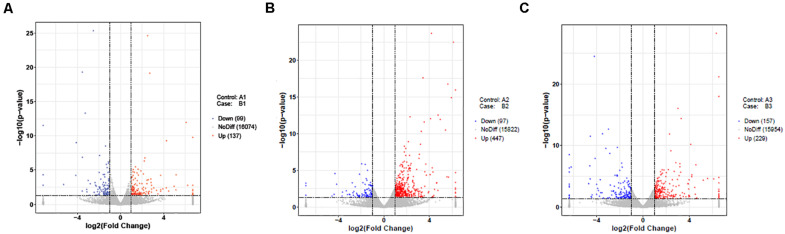
Volcano Plot of differently expressed unigenes of follicles in HR and LR chickens. The two vertical lines of dashes present twice of the difference threshold, and horizontal line of dashes indicates *p*-value $\frac{1}{2}$ of 0.05. Red dots represent significantly up-regulated genes, blue dots indicate down-regulated genes, and gray dots imply non-differentially expressed genes (*p*-value adjusted for multiple testing < 0.05). **(A)** A1 vs. B1; **(B)** A2 vs. B2; **(C)** A3 vs. B3; A1, LWF of HR hens; B1, LWF of LR hens; A2, SYF of HR hens; B2, SYF of LR hens; A3, LYF of HR hens; B3, LYF of LR hens.

In the present study, DEGs in the follicles from HR and LR hens were identified. A total of 236 DEGs out of 16,310 unigenes were found in LWF follicles, of which significantly up- and down-regulated unigenes were 137 and 99, respectively (| log2FoldChange| > 1, *P* < 0.05; Table 2, Figures 1D, 2A, and Supplementary Table 5). A total of 544 (447 up- and 97 down-regulated) and 386 unigenes (229 up- and 157 down-regulated) showed significantly differential expression in SYF and LYF follicles, respectively (| log2FoldChange| > 1, *P* < 0.05; Figures 2B,C and Supplementary Tables 6, 7). The current data indicated the number of total DEGs of SYF follicles between HR and LR hens were much greater than LWF and LYF follicles.

**TABLE 2:** Analysis of differentially expressed genes in various-sized ovarian follicles of HR and LR hens.

Follicles	Total unigenes	Nodiff.	Up-	Down-	DEGs
LWF (3.5–5.5 mm)	16310	16074	137	99	236
SYF (6–8 mm)	16366	15822	447	97	544
LYF (8.5–10.5 mm)	16340	15954	229	157	386

*No diff., non-differentially expressed unigenes (|log2FoldChange| < 1 or P > 0.05); Up-, significantly up-regulated unigenes; Down-, significantly down-regulated unigenes (|log2FoldChange| > 1, P < 0.05).*

### GO Functional Classification of DEGs

**FIGURE 3:**
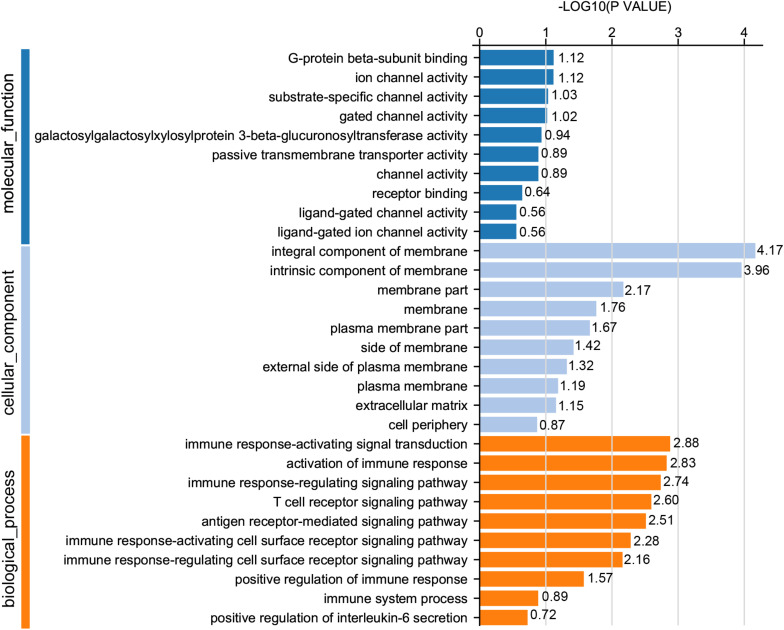
GO functional classification of DEGs in the prehierarchical follicles sized 3.5–5.5 mm in diameter of HR and LR hens. *Y*-axis: three major functional annotations of GO terms including biological process, cellular component, and molecular function. *X*-axis: the number of DEGs annotated in the group.

**FIGURE 4:**
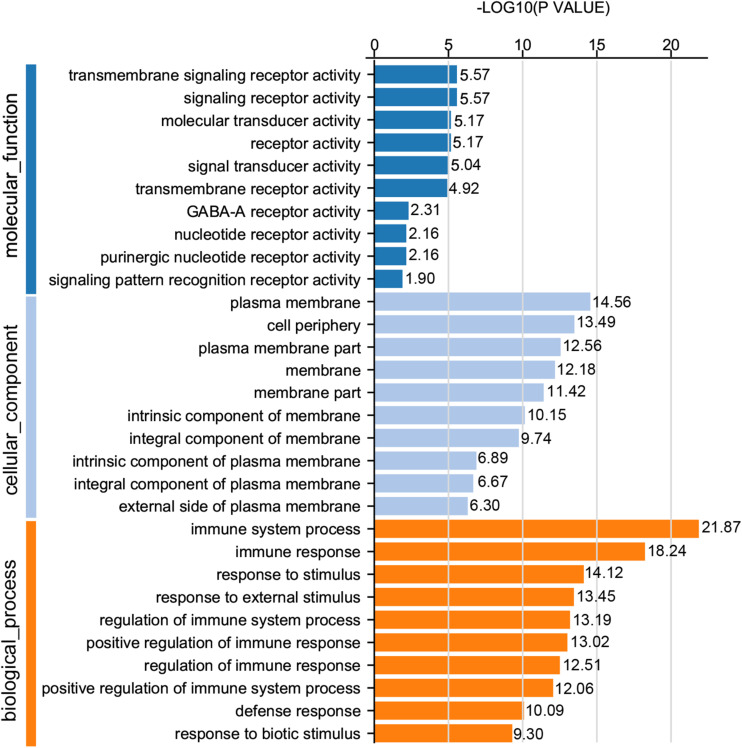
GO functional classification of DEGs in the prehierarchical follicles sized 6.0–8.0 mm in diameter of HR and LR hens. *Y*-axis: three major functional annotations of GO terms including biological process, cellular component, and molecular function. *X*-axis: the number of DEGs annotated in the group.

**FIGURE 5:**
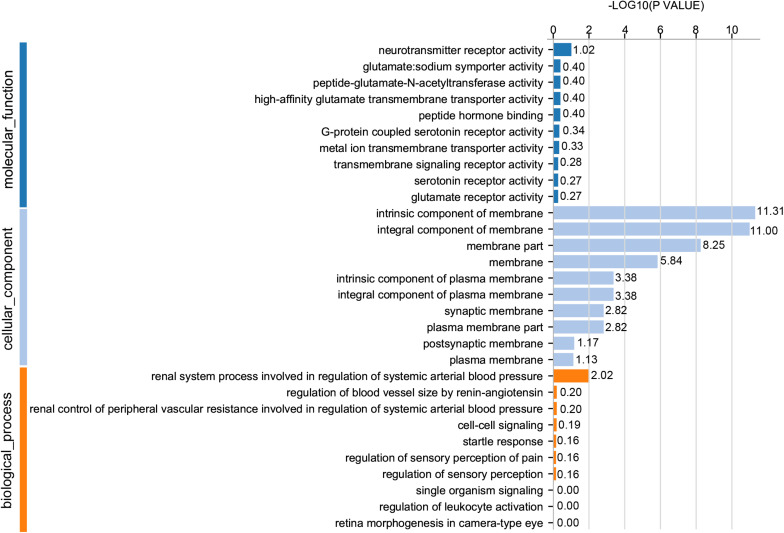
GO functional classification of DEGs in the preovulatory follicles sized 8.5–10.5 mm in diameter of HR and LR hens. *Y*-axis: three major functional annotations of GO terms including biological process, cellular component, and molecular function. *X*-axis: the number of DEGs annotated in the group.

For GO analysis, DEGs were annotated in three ontologies: biological process (BP), cellular component (CC), and molecular function (MF) of the GO database. Based upon comparison of LWF follicles between HR and LR hens, 236 DEGs were assigned to 203 different GO terms covering 162 BP, 179 CC,159 MF, and the top 10 significant GO terms for BP, CC and MF are presented in Figure 3 (*P* < 0.05, Supplementary Table 8), respectively. When comparing prehierarchical follicles (6.0–8.0 mm in diameter) of HR and LR hens, 544 DEGs were enriched to 486 different GO terms (including 404 BP, 444 CC, 375 MF) and the top 10 significant categorized GO terms are displayed in Figure 4 (*P* < 0.05, Supplementary Table 9). According to comparison of LYF follicles between HR and LR hens, 386 DEGs were grouped to 336 different GO terms (comprising 246 BP, 301 CC, 241 MF), and top 10 of the significant categorized GO terms are exhibited in Figure 5 (*P* < 0.05, Supplementary Table 10).

Comparison of significant GO terms of DEGs in the three follicle groups demonstrated the average top-10 term frequency in SYF follicles from HR and LR hens was much greater than undifferentiated LWF and preovulatory LYF (Figures 3–5). This finding indicated functional annotation data of DEG from SYF follicles clarifies their biological effects on hen egg-laying production. As shown in Figure 4, the main GO terms for SYF molecular functions included, transmembrane signaling receptor activity (GO:0004888), signaling receptor activity (GO:0038023), molecular transducer activity (GO:0060089), receptor activity (GO:0004872), signal transducer activity (GO:0004871), and transmembrane receptor activity (GO:0099600) (*P* < 0.05, Supplementary Table 9). Moreover, hypotaxis of the significant top 10 GO terms of MF was displayed in the gene ontology DAG structure (Supplementary Figure 2) and revealed that in 486 DEGs out the 12,502 GO termed unigenes by MF annotation, 375 DEGs were involved in the molecular function of GO:003674.

### Enrichment of Signaling Pathways of DEGs Annotated by GO Terms

To reveal what signaling pathways were significantly associated with egg production, and to further identify which DEGs annotated by the GO ontology were implicated in those transducing activities, enrichment analysis of the KEGG pathway was accomplished on the follicles between HR and LR hens. In LWF follicles, most relevant signaling pathways remarkably enriched consisted of cell adhesion molecules (CAM; ko 04514), PPAR signaling pathway (ko 03320), neuroactive ligand-receptor interaction (ko 04080), fatty acid biosynthesis (ko 00061), AMPK signaling pathway (ko 04152), cAMP signaling pathway (ko 04024), adipocytokine signaling pathway (ko 04920), ECM-receptor interaction (ko 04512), p53 signaling pathway (ko 04115), and insulin secretion (ko 04911). Collectively, there were 44 DEGs (24 up- and 20 down-regulated) to be significantly enriched in these 10 KEGG pathways (Supplementary Table 11).

In SYF follicles, the prominently enriched KEGG pathways included phagosome (ko 04145), CAMs (ko 04514), the NF-kappa B signaling pathway (ko 04064), the calcium signaling pathway (ko 04020), the Toll-like receptor signaling pathway (ko 04620), neuroactive ligand-receptor interaction (ko 04080), and the GABAergic synapse (ko 04727). In total, 87 DEGs (80 up- and 7 down-regulated) were implicated in the seven signaling pathways (Supplementary Table 12).

In LYF follicles, there were 11 relevant KEGG pathways encompassing the neuroactive ligand-receptor interaction (ko 04080), CAMs (ko 04514), circadian entrainment (ko 04713), glutamatergic synapse (ko 04724), mucin type *O*-glycan biosynthesis (ko 00512), the PPAR signaling pathway (ko 03320), D-arginine and D-ornithine metabolism (ko 00472), carbohydrate digestion and absorption (ko 04973), the JAK-STAT signaling pathway (ko 04630), the cAMP signaling pathway (ko 04024), and cytokine-cytokine receptor interactions (ko 04060). Totally, 71 DEGs (57 up- and 14 down-regulated) were encircled in the 11 signaling pathways (Supplementary Table 13).

Obviously, there were two co-enriched pathways (CAMs and neuroactive ligand-receptor interaction) in LWF, SYF, and LYF follicles and two co-enriched pathways (PPAR signaling pathway and cAMP signaling pathway) in LWF and LYF follicles of HR and LR hen ovaries. The relevant up-regulated DEGs included *ACSBG1*, *CNTN1*, *DMB2*, *FABP7*, *GRIK1*, *GABRA1*, *GABRB2*, *GABRG1*, *GABRQ*, *GALR2*, *GRIA2*, *GRIA4*, *GRIN2A*, *HRH1*, *HRH2*, *HTR1A*, *HTR2A*, *ITGB2*, *LEPR*, *LRRC4C*, *NCAM1*, *NLGN1*, *NRCAM*, *PIK3R1*, *PRLR*, *PTPRC*, *P2RX7*, *RYR2*, *VCAM1*, *VCAN*, *VIP*, and *VIPR2*, et al. (Supplementary Tables 11–13). The related down-regulated DEGs comprised *ACOX2*, *CCKBR*, *CDKN1A*, *CHRNA4*, *CREB3L3*, *FABP3*, *F2RL1*, *GRPR*, *MC2R*, *NCAM2*, *NRXN1*, *OPRD1*, *P2RX1*, *RBPJL*, and *SSTR2*, et al. (Supplementary Tables 11–13).

### Characterization of the Potential, Critical DEGs Involved in Egg-Laying Production

**FIGURE 6:**
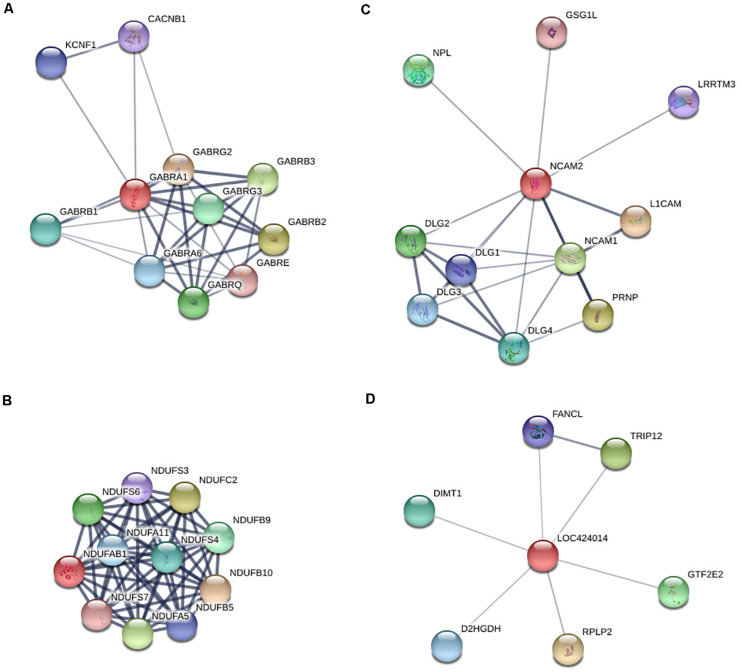
Predicted protein to protein interaction network of the four co-expressed DEGs in LWF, SYF and LYF follicles of HR and LR hens. **(A)** Presumed regulatory network of *GABRA1* in neuroactive ligand-receptor interaction pathway by using the STRING database; **(B)** Inferred interaction network of *NDUFAB1* in oxidative phosphorylation pathway; **(C)** Deduced regulatory network of *NCAM2* in cell adhesion molecules interaction pathway; **(D)** Predicted interaction network of *LOC424014* in putative methyltransferase.

To further explore critical candidate genes potentially involved in high and low egg-laying chickens, 47 co-expressed DEGs in LWF and SYF follicles of HR and LR hens were identified (Figure 1D); among them, 30 known genes are listed in Table S14 including *P2RX1*, *CAB39L*, *BLK*, *CSMD3*, *GPR65*, and *ADRB2* (Table 3). Sixty-eight co-expressed DEGs of SYF and LYF follicles were enriched (Figure 1D) and 42 of known genes were focused on (Supplementary Table S15) including *CSMD1*, *PLPP4*, *ATF3*, *PRLL*, *STMN3*, and *RORB* (Table 3). In 54 co-expressed DEGs in LWF and LYF follicles, 35 of known genes were concentrated on (Figure 4 and Supplementary Table 16) and the most relevant genes included *PIK3R1*, *PERP1*, *ACSBG1*, *MRTO4*, *CDKN1A*, and *EDA2R* (Table 3). Moreover, there were 13 co-expressed DEGs obtained in LWF, SYF, and LYF follicles of HR and LR hens (Figure 4 and Supplementary Table 17). Among them, three known genes, one novel gene, and four uncharacterized genes related to egg production were selected from the co-expressed DEGs, the characterized genes included *GABRA1*, *NDUFAB1*, *NCAM2*, *LOC424014* (Table 3). In order to lay a useful molecular foundation for further elucidating the genetic mechanism underlying ovarian follicle development associated with egg production in chicken, a potential physical interaction network of proteins encoded by four genes as shown in Figure 6. Additionally, the uncharacterized genes in this study comprised of ENSGALG00000033498, ENSGALG00000036119, ENSGALG00000037683, and ENSGALG00000046112 (Supplementary Table 17).

**TABLE 3:** Characterization of key co-expressed DEGs in LWF, SYF, and LYF follicles of HR and LR hens.

Samples	Genes	RP (H1 vs. L1)	RP (H2 vs. L2)	RP (H3 vs. L3)	KEGG Pathway	Britehierarchy (KO)	UniProtID	PFAM Domains	eggNOG
LWF&SYF	*P2RX1*	Up	Up	–	Neuroactive ligand-receptor interaction	gga04080	Q8AWC0	PF00864	ENOG410IFJF
	*CAB39L*	Up	Up	–	mTOR signaling pathway	gga04150	F1NV80	PF08569	KOG1566
	*BLK*	Up	Up	–	Non-receptor tyrosine kinases	gga422035	E1BYL7	PF07714	KOG0197
	*CSMD3*	Up	Up	–	Phosphatase activity	gga01009	–	PF00084	–
	*GPR65*	Up	Up	–	GPCR ligand binding	gga04030	F1NJ83	PF00001	KOG3656
	*ADRB2*	Up	Up	–	Neuroactive ligand-receptor interaction	gga04080	A0A1D5P7A9	PF00001	–
SYF&LYF	*CSMD1*	–	Up	Up	Phosphatase activity	gga01009	–	PF00084	–
	*PLPP4*	–	Up	Up	Glycerolipid metabolism	gga00561	R4GJ86	PF01569	KOG3030
	*ATF3*	–	Up	Up	cyclic AMP-dependent transcription	gga03000	A0A3Q3B2P0	PF00170	–
	*PRLL*	–	Down	Down	Prolactin signaling pathway	gga04917	C6ZDB7	PF00103	ENOG410II74
	*STMN3*	–	Down	Down	Regulation of Rac signal transduction	gga04131	O93388	PF 00836	ENOG410IEAZ
	*RORB*	–	Down	Up	Circadian rhythm	gga04710	Q98934	PF00104	KOG4216
LWF&LYF	*PIK3R1*	Up	–	Up	PI3K-Akt signaling pathway	gga427171	A0A1D5PUK7	PF16454	–
	*PERP1*	Up	–	Up	p53 signaling pathway	gga421683	R4GHP1	PF00822	KOG4671
	*ACSBG1*	Up	–	Up	Fatty acid biosynthesis	gga00061	F1NLD6	PF00501	KOG1256
	*MRTO4*	Down	–	Down	Ribosome biogenesis	gga03009	F1P353	PF00466	KOG0816
	*CDKN1A*	Down	–	Down	ErbB signaling pathway	gga04012	Q8AYE7	PF02234	–
	*EDA2R*	Down	–	Down	Cytokine-cytokine receptor interaction	gga04060	A2SWM7	PF00020	–
LWF&SYF &LYF	*GABRA1*	Up	Up	Up	Neuroactive ligand-receptor interaction	gga04080	P19150	PF02931	KOG3642
	*NDUFAB1*	Down	Down	Down	Oxidative phosphorylation	gga00190	E1C4C0	PF00550	KOG1748
	*NCAM2*	Down	Down	Down	Cell adhesion molecules	gga04514	–	PF13895	–
	*LOC424014*	Down	Up	Down	putative methyltransferase	gga424014	A0A1D5P5L5	PF08241	–

*H1 vs. L1, indicating DEGs expressed in LWF follicles of HR and LR hen ovaries; H2 vs. L2, DEGs in SYF follicles of HR and LR; H3 vs. L3, DEGs in LYF follicles of HR and LR; Genes, co-expressed DEGs in the samples; RP, regulation pattern; KO, KEGG Orthology.*

### Validation of the Selected DGEs by RT-qPCR

**FIGURE 7:**
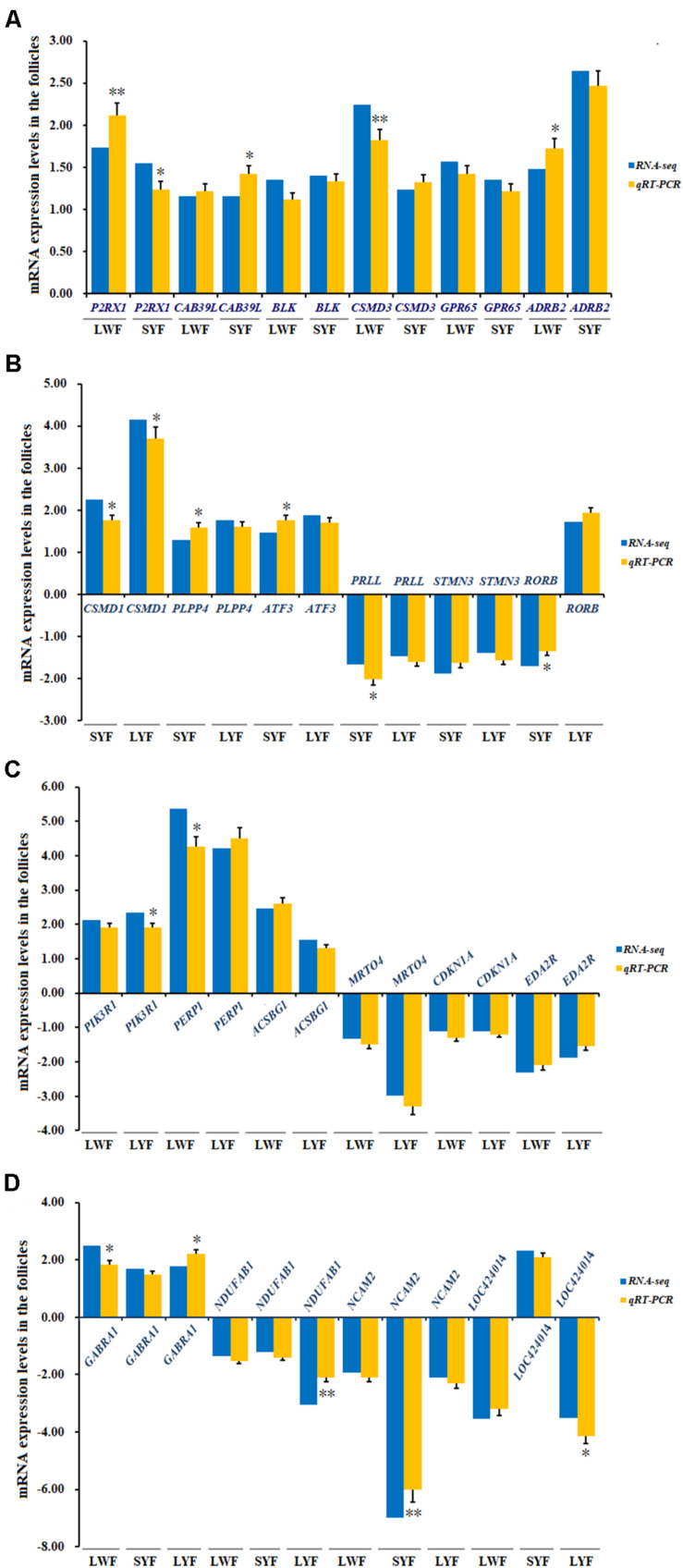
Evaluation and comparison of mRNA expression levels (Log2FC) of 22 candidate genes when analyzed by RNA Seq and quantitative RT-qPCR. **(A)** Relative expression levels of the DEG mRNA co-expressed in LWF and SYF; **(B)** Relative expression levels of the DEG mRNA co-expressed in SYF and LYF; **(C)** Relative expression levels of the DEG mRNA co-expressed in LWF and LYF; **(D)** Relative expression levels of the DEG mRNA co-expressed in LWF and SYF and LYF. The relative expression level of each gene was quantified by the 2^−ΔΔCT^ method and *18S rRNA* was used as the internal control for normalization of the results.

After the aforementioned analyses, 22 of most relevant DGEs in the Table 3 were selected for validation by RT-qPCR. As shown in Figure 7, all of the six co-expressed DGEs (*P2RX1*, *CAB39L*, *BLK*, *CSMD3*, *GPR65*, and *ADRB2*) were up-regulated in LWF and SYF follicles sampled from HR and LR hens. For the candidate genes from SYF and LYF follicles, three genes (*CSMD1*, *PLPP4*, and *ATF3*) were up-regulated and two genes (*PRLL* and *STMN3*) were down-regulated at the different development stages examined, but candidate *RORB* was down-regulated at the SYF stage, while up-regulated at the LYF stage. For the candidate genes co-expressed in LWF and LYF follicles, three genes (*PIK3R1*, *PERP1*, and *ACSBG1*) were up-regulated; however, three genes (*MRTO4*, *CDKN1A* and *EDA2R*) were down-regulated. For the DGEs co-expressed in LWF, SYF and LYF follicles, one gene (*GABRA1*) was up-regulated, two genes (*NDUFAB1* and *NCAM2*) were down-regulated, and a novel gene (*LOC424014*) was down-regulated in the LWF and LYF follicles, while it was up-regulated in the SYF group. Our results show the significantly differential expression levels of the most transcripts detected by RT-qPCR analysis were consistent with the observations determined by RNA-seq (Figure 7). While there are significant differences in the expression levels of the other genes, including *P2RX1*, *CAB39L*, *CSMD1*, *CSMD3*, *PRLL*, *NDUFAB1*, *NCAM2*, et al. the observed expression trends coordinate with the RNA-seq results, confirming the transcriptome sequencing results are reliable, and can be further studied for elucidating the genetic mechanism underlying ovarian follicle development associated with egg production in chicken.

## Discussion

The ovary is known as one of the most important reproductive organs in adult poultry, which contains many follicles at various developmental stages. Therefore, various regulatory mechanisms in addition to physical contact mediate follicular interactions and directly contribute to egg-laying production (Komatsu and Masubuchi, 2016). The follicle is the major ovarian compartment which enables the ovary to fulfill its dual function of gametogenesis and steroidogenesis (Qin et al., 2015). The various-size growing follicles are mainly composed of oocytes, one or more than two rows of granulosa cells (GCs) surrounding the oocyte, and a theca cell layers at the exterior of the GCs, marking the basement membrane (Johnson and Woods, 2009; Qin et al., 2015). The theca cell layer not only serves as the target apparatus for action of gonadotrophins (FSH and LH) of the hypothalamic-pituitary-gonadal axis that controls the whole reproductive system (Padmanabhan et al., 2002; Woods and Johnson, 2005; Johnson, 2015b), but also acts as a key reproductive moderator and effector to dominate development of oocytes, proliferation and differentiation of granulosa and theca cells, and steroid synthesis (progesterone, estradiol, and testosterone) and secretion (Channing et al., 1980; Richards and Ascoli, 2018). Moreover, a myriad of local autocrine factors and relevant proteins (especially receptors, such as FSHR, ESRs, AMHRs, and VIPR) are synthesized in response to endocrine hormones, paracrine factors, and extra- and intra-ovarian signaling stimuli (Lovell et al., 2003; Xu et al., 2018; Zhang et al., 2019; Mishra et al., 2020). Particularly, genetic regulation of 6.0–8.0 mm diameter follicles are generally involved in follicle selection (Johnson and Woods, 2009; Son et al., 2011; Johnson, 2015b) and may possess an exclusive influence on hierarchy of undifferentiated prehierarchical follicles. Obviously, follicles at various developmental stages have their own distinct molecular biological characteristics and play different roles in contributing to ovary growth and development. Therefore, identification of DEGs of various-sized follicles that differ between HR and LR hens will identify potential candidate genes that control egg production traits. Moreover, our data can provide evidence for highlighting the molecular mechanisms underlying their involvement in formation of egg production traits. Additionally, intensively mining the valuable molecular markers related to egg-laying traits can facilitate breeding programs aiming to improve egg productivity in chickens.

In this study, high-throughput transcriptome technology was performed to analyze and identify DEGs of the follicles sized 3.5–5.5, 6.0–8.0, and 8.5–10.5 mm in diameter between the LB and JB chicken breeds. To our knowledge, this is the first study to highlight potential pivotal genes associated with high and low rates of egg production in hen ovarian follicles at various developmental stages via RNA sequencing analysis. Our data showed 47 co-expressed DEGs in LWF and SYF follicles, 68 co-expressed DEGs in SYF and LYF, and 54 co-expressed DEGs in LWF and LYF. These DEGs were significantly related with various physiological functions of follicle growth and development, which have potential to be involved in hen egg-laying production. Moreover, by further enrichment and analyses of the DEGs, 13 of the co-expressed DEGs were obtained in LWF, SYF, and LYF follicles of HR and LR hens. Among them, three known genes (*GABRA1*, *NDUFAB1*, and *NCAM2*) and one novel gene (*LOC424014*) were characterized.

The *GABRA1* gene encodes γ-aminobutyric acid type A receptor alpha1 subunit (GABRA1), which is one member of the gamma-aminobutyric acid signaling pathway of the PANTHER subfamily (PTHR18945:SF514). Previously, studies demonstrated the γ-aminobutyric acid type A receptor (GABA-A receptors, GABAR) is a ligand-gated Cl^–^ ion channel (Morrow et al., 1990), mainly comprising two α subunits, two β subunits, and either one γ or δ subunit (Barrera et al., 2008; Chuang and Reddy, 2018). This configuration shows the receptor’s vital role in apoptosis regulations, mitochondrial function, inhibitory effects of different neurotransmitters, and synaptic plasticity by interaction with neuroactive steroids such as androstane and progesterone derivatives (Tuem and Atey, 2017). Although the exact roles of GABRA1 and the GABAR in ovarian follicular function remain unclear in chicken, a study reported the inhibitory effect of xanthohumol on evoked glutamate release was antagonized by suppressing the GABAR/Ca^2+^-calmodulin/AC/cAMP/PKA cascade in rat hippocampus (Chang et al., 2016). Moreover, the AC/cAMP/PKA pathway was involved in granulosa cell proliferation and differentiation of ovarian follicles in hen (Dupont et al., 2012; Nemer et al., 2018). In this study, our results revealed that transcript levels of *GABRA1* were remarkably up-regulated in all sizes of ovarian follicles from LR hens compared to HR hens. Therefore, up-regulated *GABRA1* gene may be closely related with low hen egg-laying production. This was also supported by the result that expression levels of *GABRA1* mRNA were down-regulated in goose ovaries during the laying period when compared with the non-laying period (Luan et al., 2014).

The *NDUFAB1* gene encodes the acyl carrier protein (ACP), NDUFAB1 subunit of mitochondrial complex I (NADH: ubiquinone oxidoreductase), which is involved in cytosolic and mitochondrial fatty acid synthesis (FAS) pathways. The active form, holo-ACP, serves as a FAS platform, using its 4′-phosphopantetheine group to present covalently attached FAS intermediates to enzymes responsible for the acyl chain elongation process (Masud et al., 2019). Down-regulation of mitochondrial ACP expression in HEK293T cells has brought about a slowing of cell growth rate or a high level of cell death (Feng et al., 2009). Moreover, NDUFAB1 is linked directly to mitochondrial ATP production, indirectly to potential reactive oxygen species (ROS) generation (Pecorelli et al., 2013), and reduced mitochondrial ATP production directly resulted in increased cell apoptosis (Yu et al., 2013).

Neural cell adhesion molecule 2 (NCAM2), encoded by the *NCAM2* gene, was originally reported to be a key regulator of cell adhesion, axon growth, and fasciculation (Maness and Schachner, 2007). It was also shown to play a critical role in the regulation of granulosa cell differentiation and promoted the development of the cumulus cell phenotype (Eppig et al., 1997; Kaese et al., 2015). In this study, transcript levels of *NDUFAB1* and *NCAM2* were significantly down-regulated in the ovarian follicles of all size categories from LR hens compared to HR hens. This result suggested down-regulated expression of *NDUFAB1* and *NCAM2* transcripts may be strongly associated with hen low egg-laying production.

The *LOC424014* gene encodes a putative methyltransferase that interacts with histone modifications to catalyze DNA methylation to inhibit gene transcription and induce gene silencing (Bird, 2002; Court et al., 2019). This is an uncharacterized novel gene in chicken, which was identified to be significantly down-regulated in the ovarian follicles of 3.5–5.5 and 8.5–10.5 mm in diameters, but up-regulated in 6.0–8.0 mm diameter ovarian follicles of LR hens compared to HR hens. Undoubtedly, these four candidate genes are most potentially associated with egg production traits in chicken, although further studies need to be conducted to confirm this hypothesis. Additionally, the other 18 selected DEGs validated by RT-qPCR, including *P2RX1*, *CAB39L*, *BLK*, *CSMD3*, *GPR65*, *ADRB2*, *CSMD1*, *PLPP4*, *ATF3*, *PRLL*, *STMN3*, *RORB*, *PIK3R1*, *PERP1*, *ACSBG1*, *MRTO4*, *CDKN1A*, and *EDA2R* were significantly differentially expressed in ovarian follicles at the various development stages examined, which indicates they may also be promising molecular biomarkers of chicken egg production.

In this study, four signaling pathways including CAMs, the neuroactive ligand-receptor interaction, the PPAR signaling pathway and the cAMP signaling pathway were found to be the most important pathways associated with high and low egg production. Among them, the CAMs pathway was previously reported to mediate many biologic actions such as cell to cell recognition, cell to matrix adhesion, and in early vertebrae embryos (Li et al., 2007). In the current pathway, 15 candidate DEGs including *CDKN1A*, *CNTN1*, *CREB3L3*, *DMB2*, *ITGB2*, *LRRC4C*, *NCAM1*, *NCAM2*, *NLGN1*, *NRCAM*, *NRXN1*, *PTPRC*, *RBPJL*, *VCAM1*, and *VCAN* were initially identified to be involved in ovarian follicle development. Previous studies demonstrated the neuroactive ligand-receptor interaction may be the most important pathway contributing to differential egg production rates between HR and LR hens (Tao et al., 2017; Zhang et al., 2019). In the present study, this pathway was enriched to be involved in chicken ovarian follicle development, of which 47 DEGs including *CCKBR*, *CHRNA4*, *F2RL1*, *GABRA1*, *GABRB2*, *GABRG1*, *GABRQ*, *GALR2*, *GRIA2*, *GRIA4*, *GRIK1*, *GRIN2A*, *GRPR*, *HRH1*, *HRH2*, *HTR1A*, *HTR2A*, *LEPR*, *MC2R*, *OPRD1*, *PRLR*, *P2RX1*, *P2RX7*, *SSTR2*, *VIP*, and *VIPR2* were implicated.

Previous studies proved the PPAR signaling pathway plays an important role in ovarian follicle development and normal ovarian function by being indirectly involved in oocyte maturation and ovulation via regulation of steroid hormone synthesis in granulosa cell (Dupont et al., 2012). In the hypothalamus/pituitary gland of laying hens, peroxisome proliferator-activated receptor A (PPARA) and prostaglandin-D synthetase (PGDS) mRNA levels were highly correlated to high egg production in L2 Taiwan country chickens (Chen et al., 2010). In this study, seven DEGs consisting of *ACOX2*, *ACSBG1*, *ACSBG2*, *FABP3*, *FABP7*, *RXRG*, and *SCD* of the PPAR pathway were found to be co-expressed in LWF and LYF follicles of HR and LR hen ovaries. Moreover, it is well-known that FSH acts with its receptor FSHR in an endocrine dependent manner and plays an essential role in the reproductive system including steroidogenesis, folliculogenesis, and follicular maturation (Choi et al., 2005; Johnson, 2015a). FSHR, a member of the superfamily of G-protein-coupled receptors, is exclusively expressed on granulosa cells of ovarian follicles and mediates FSH signal transduction through cAMP signaling pathway (Simoni et al., 1997; Johnson and Woods, 2009; Johnson, 2015b). cAMP signaling provokes a variety of processes required for growth, selection, differentiation, and maturation of ovarian follicles (Dupont et al., 2012; Richards and Ascoli, 2018). In the current study, 10 DEGs consisting of *CREB3L3*, *GRIA2*, *GRIA4*, *GRIN2A*, *HTR1A*, *MC2R*, *PIK3R1*, *RYR2*, *SSTR2*, and *VIPR2* were discovered to be involved in this signaling pathway to control of granulosa cell proliferation and apoptosis, oocyte meiosis and maturation, follicular differentiation and atresia, and secretion of gonadotrophin-release hormone via crosstalk or intracellular interactions with several pathways. These pathways include PPAR signaling, neuroactive ligand-receptor interaction, the calcium signaling pathway, the GnRH signaling pathway, and the PI3K/AKT signaling pathway (Chang et al., 2016; Nemer et al., 2018; Richards and Ascoli, 2018; Liu et al., 2019). Taken together, our present data provide additional evidence for supporting these four signaling pathways were related to chicken egg production.

In summary, this study represents the first transcriptome analysis of hen ovarian follicles at different developmental stages between chickens that possess high and low rates of egg production. It established a foundation for further investigation of regulatory mechanisms of the candidate genes involved in ovary follicular development, for understanding the molecular regulation of egg-laying traits, and discovering potential molecular markers that can facilitate chicken breeding programs to enhance egg-laying productivity.

## Data Availability Statement

Our raw RNA sequencing data is available at BioProject under accession numbers PRJNA669967 and PRJNA670553.

## Ethics Statement

All procedures executed in chickens were approved by the Institutional Animal Care and Use Committee (IACUC) of Jilin Agricultural University (Changchun, China). All birds were sacrificed prior to the removal of organs based upon the IACUC Guidelines for the euthanasia of experimental animals [Permission No. GR (J) 19-030].

## Author Contributions

XC, XS, and RX conceived and designed the experiments. XC, XS, IC, NQ, XX, SL, and RX performed the experiments. XC, RX, XX, and JG analyzed the data. XC and RX wrote the manuscript. JG reviewed and edited the manuscript. All the authors contributed to the article and approved the submitted version.

## Conflict of Interest

The authors declare that the research was conducted in the absence of any commercial or financial relationships that could be construed as a potential conflict of interest.

## Footnotes

1 http://en.wikipedia.org/wiki/FASTQ_format

2 https://www.ncbi.nlm.nih.gov/assembly/GCF_000002315.6

3 https://sourceforge.net/projects/mev-tm4/files/latest/download

4 http://cran.r-project.org/web/packages/car/index.html

5 http://geneontology.org/

6 http://www.bioconductor.org/packages/3.0/bioc/html/topGO.html

7 http://www.kegg.jp/

8 http://www.reactome.org

9 http://www.genome.jp/kegg/

10 http://www.pantherdb.org

11 http://string-db.org/

12 http://www.uniprot.org/help/uniprotkb

13 http://enzyme.expasy.org/

14 http://eggnog.embl.de/version_3.0/

15 http://asia.ensembl.org/Gallus_gallus/Info/Index

## Supplementary Material

Supplementary Figure 1 Characterization of ovarian follicular morphology in the laying hens sampled. a, White follicles 3.5–5.5 mm in diameter were classified as large white follicles (LWF). b, Yellow follicles 6.0–8.0 mm were grouped as small yellow follicles (SYF). The hierarchical follicles were categorized as F6, F5… and F1 according to their diameters from the smallest to the largest, in which the follicle F6 were collected to yellow follicles 8.5–10.5 mm, and was sampled as large yellow follicles (LYF) in this study.

Supplementary Figure 2 The gene ontology DAG structure for hypotaxis of the significant top 10 GO terms of molecular function.

Supplementary Table 1 Quality of sequenced raw data.

Supplementary Table 2 Cleaned data of filtered reads.

Supplementary Table 3 Results of the RNASeq map.

Supplementary Table 4 Results of the RNASeq mapped events.

Supplementary Table 5 DEGs in the LWF of HR and LR hens (H1 vs. L1).

Supplementary Table 6 DEGs in the SYF of HR and LR hens (H2 vs. L2).

Supplementary Table 7 DEGs in the LYF of HR and LR hens (H3 vs. L3).

Supplementary Table 8 GO terms of DEGs in LWF of HR and LR hens.

Supplementary Table 9 GO terms of DEGs in SYFF of HR and LR hens.

Supplementary Table 10 GO terms of DEGs in LYF of HR and LR hens.

Supplementary Table 11 Enriched KEGG pathways and DEGs in ovarian LWF follicles of HR and LR hens.

Supplementary Table 12 Enriched KEGG pathways and DEGs in ovarian SYF follicles of HR and LR hens.

Supplementary Table 13 Enriched KEGG pathways and DEGs in ovarian LYF follicles of HR and LR hens.

Supplementary Table 14 Co-expressed DEGs in LWF and SYF follicles of HR and LR hens.

Supplementary Table 15 Co-expressed DEGs in SYF and LYF follicles of HR and LR hens.

Supplementary Table 16 Co-expressed DEGs in LWF and LYF follicles of HR and LR hens.

Supplementary Table 17 Co-expressed DEGs in LWF, SYF, and LYF follicles of HR and LR hens.

## References

[B1] Ashburner M. Ball C. A. Blake J. A. Botstein D. Butler H. Cherry J. M. (2000). Gene ontology: tool for the unification of biology. The Gene Ontology Consortium. *Nat. Genet.* 25 25–29. 10.1038/75556 10802651 PMC3037419

[B2] Barrera N. P. Betts J. You H. Henderson R. M. Martin I. L. Dunn S. M. (2008). Atomic force microscopy reveals the stoichiometry and subunit arrangement of the αarre GABAA receptor. *Mol. Pharmacol.* 73 960–967. 10.1124/mol.107.042481 18079275

[B3] Bird A. (2002). DNA methylation patterns and epigenetic memory. *Genes Dev.* 16 6–21. 10.1101/gad.947102 11782440

[B4] Chang Y. Lin T. Y. Lu C. W. Huang S. K. Wang Y. C. Wang S. J. (2016). Xanthohumol-induced presynaptic reduction of glutamate release in the rat hippocampus. *Food Funct.* 7 212–226. 10.1039/c5fo01005e 26667007

[B5] Channing C. P. Schaerf F. W. Anderson L. D. Tsafriri A. (1980). Ovarian follicular and luteal physiology. *Int. Rev. Physiol.* 22 117–201. 6248477

[B6] Chen G. H. Wang K. H. Wang J. Y. Ding C. Yang N. (2004). *Poultry genetic resources in China (in Chinese).* Shanghai: Shanghai Scientific & Technical Publisher, China.

[B7] Chen L. R. Lee S. C. Lin Y. P. Hsieh Y. L. Chen Y. L. Yang J. R. (2010). Prostaglandin-D synthetase induces transcription of the LH beta subunit in the primary culture of chicken anterior pituitary cells via the PPAR signaling pathway. *Theriogenology* 73 367–382. 10.1016/j.theriogenology.2009.09.020 19954828

[B8] Choi J. H. Choi K. C. Auersperg N. Leung P. C. K. (2005). Gonadotropins upregulate the epidermal growth factor receptor through activation of mitogen-activated protein kinases and phosphatidyl-inositol-3-kinase in human ovarian surface epithelial cells. *Endocr. Relat. Cancer* 12 407–421. 10.1677/erc.1.00896 15947112

[B9] Chuang S. H. Reddy D. S. (2018). Genetic and molecular regulation of extrasynaptic GABA-A receptors in the brain: therapeutic insights for epilepsy. *J. Pharmacol. Exp. Ther.* 364 180–197. 10.1124/jpet.117.244673 29142081 PMC5771312

[B10] Court F. Le Boiteux E. Fogli A. Müller-Barthélémy M. Vaurs-Barrière C. Chautard E. (2019). Transcriptional alterations in glioma result primarily from DNA methylation-independent mechanisms. *Genome Res.* 29 1605–1621. 10.1101/gr.249219.119 31533980 PMC6771409

[B11] Dupont J. Reverchon M. Cloix L. Froment P. Ramé C. (2012). Involvement of adipokines, AMPK, PI3K and the PPAR signaling pathways in ovarian follicle development and cancer. *Int. J. Dev. Biol.* 56 959–967. 10.1387/ijdb.120134jd 23417417

[B12] Eppig J. J. Chesnel F. Hirao Y. O’Brien M. J. Pendola F. L. Watanabe S. (1997). Oocyte control of granulosa cell development: how and why. *Hum. Reprod.* 12 127–132. 9433969

[B13] Etches R. J. Kelly J. D. Anderson-Langmuir C. E. Olson D. M. (1990). Prostaglandin production by the largest preovulatory follicles in the domestic hen (Gallus domesticus). *Biol. Reprod.* 43 378–384. 10.1095/biolreprod43.3.378 2125506

[B14] Feng D. Witkowski A. Smith S. (2009). Down-regulation of mitochondrial acyl carrier protein in mammalian cells compromises protein lipoylation and respiratory complex I and results in cell death. *J. Biol. Chem.* 284 11436–11445. 10.1074/jbc.M806991200 19221180 PMC2670149

[B15] Johnson A. L. (2015a). “Chapter 28: reproduction in the Female,” in . *Sturkie’s Avian Physiology*, 6th Edn, ed. Scanes C. G. (New York, NY: Academic Press, USA).

[B16] Johnson A. L. (2015b). Ovarian follicle selection and granulosa cell differentiation. *Poult. Sci*. 94 781–785. 10.3382/ps/peu008 25535403

[B17] Johnson A. L. Woods D. C. (2009). Dynamics of avian ovarian follicle development: cellular mechanisms of granulosa cell differentiation. *Gen. Comp. Endocrinol.* 163 12–17. 10.1016/j.ygcen.2008.11.012 19059411

[B18] Johnson P. (2012). Follicle selection in the avian ovary. *Reprod. Domest. Anim.* 47(Suppl. 4), 283–287. 10.1111/j.1439-0531.2012.02087.x 22827382

[B19] Kaese M. Galuska C. E. Simon P. Braun B. C. Cabrera-Fuente S. H. A. Middendorff R. (2015). Polysialylation takes place in granulosa cells during apoptotic processes of atretic tertiary follicles. *FEBS J.* 282 4595–4606. 10.1111/febs.13519 26392163

[B20] Kang B. Guo J. R. Yang H. M. Zhou R. J. Liu J. X. Li S. Z. (2009). Differential expression profiling of ovarian genes in prelaying and laying geese. *Poult. Sci.* 88 1975–1983. 10.3382/ps.2008-00519 19687284

[B21] Kim D. Paggi J. M. Park C. Bennett C. Salzberg S. L. (2019). Graph-based genome alignment and genotyping with HISAT2 and HISAT-genotype. *Nat. Biotechnol.* 37 907–915. 10.1038/s41587-019-0201-4 31375807 PMC7605509

[B22] Komatsu K. Masubuchi S. (2016). Observation of the dynamics of follicular development in the ovary. *Reprod. Med. Biol.* 16 21–27. 10.1002/rmb2.12010 29259446 PMC5715870

[B23] Li L. Zhu Q. Lu Y. (2007). Cell adhesion molecules and metastasis. *Chin. Pharmacol. Bull.* 23 568–571. 10.1631/jzus.2007.B0566 17657858 PMC1934951

[B24] Lin P. Rui R. (2010). Effects of follicular size and FSH on granulosa cell apoptosis and atresia in porcine antral follicles. *Mol. Reprod. Dev.* 77 670–678. 10.1002/mrd.21202 20652999

[B25] Lin J. B. Chen X. Liu M. D. (2002). *SPSS 11.0 Statistical Analysis Actual Practice Design (in Chinese).* Beijing: Chinese Railroad Publishing House, China.

[B26] Liu Y. X. Zhang Y. Li Y. Y. Liu X. M. Wang X. X. Zhang C. L. (2019). Regulation of follicular development and differentiation by intra-ovarian factors and endocrine hormones. *Front. Biosci.* 24:983–993. 10.2741/4763 30844725

[B27] Lohmann Tierzucht GmBH (2005). *Management Guide for Laying Hens: Lohmann Brown-Classic.* Cuxhaven: Lohmann Tierzucht GmbH.

[B28] Lovell T. M. Gladwell R. T. Groome N. P. Knight P. G. (2003). Ovarian follicle development in the laying hen is accompanied by divergent changes in inhibin A, inhibin B, activin A and follistatin production in granulosa and theca layers. *J. Endocrinol.* 177 45–55. 10.1677/joe.0.1770045 12697036

[B29] Luan X. Liu D. Cao Z. Luo L. Liu M. Gao M. (2014). Transcriptome profiling identifies differentially expressed genes in Huoyan goose ovaries between the laying period and ceased period. *PLoS One* 9:e113211. 10.1371/journal.pone.0113211 25419838 PMC4242529

[B30] Maness P. F. Schachner M. (2007). Neural recognition molecules of the immunoglobulin superfamily: signaling transducers of axon guidance and neuronal migration. *Nat. Neurosci.* 10 19–26. 10.1038/nn1827 17189949

[B31] Masud A. J. Kastaniotis A. J. Rahman M. T. Autio K. J. Hiltunen J. K. (2019). Mitochondrial acyl carrier protein (ACP) at the interface of metabolic state sensing and mitochondrial function. *Biochem. Biophys. Acta. Mol. Cell Res.* 1866:118540. 10.1016/j.bbamcr.2019.118540 31473256

[B32] Mishra S. K. Chen B. Zhu Q. Xu Z. Ning C. Yin H. (2020). Transcriptome analysis reveals differentially expressed genes associated with high rates of egg production in chicken hypothalamic-pituitary-ovarian axis. *Sci. Rep.* 10:5976. 10.1038/s41598-020-62886-z 32249807 PMC7136225

[B33] Morrow A. L. Pace J. R. Purdy R. H. Paul S. M. (1990). Characterization of steroid interactions with gamma-aminobutyric acid receptor-gated chloride ion channels: evidence for multiple steroid recognition sites. *Mol. Pharmacol.* 37 263–270. 1689453

[B34] Nemer A. Azab A. N. Rimon G. Lamprecht S. Ben-Menahem D. (2018). Different roles of cAMP/PKA and PKC signaling in regulating progesterone and PGE2 levels in immortalized rat granulosa cell cultures. *Gen. Comp. Endocrinol.* 269:8895. 10.1016/j.ygcen.2018.08.019 30144443

[B35] Onagbesan O. Bruggeman V. Decuypere E. (2009). Intra-ovarian growth factors regulating ovarian function in avian species: a review. *Anim. Reprod. Sci.* 111 121–140. 10.1016/j.anireprosci.2008.09.017 19028031

[B36] Padmanabhan V. Karsch F. J. Lee J. S. (2002). Hypothalamic, pituitary and gonadal regulation of FSH. *Reprod. Suppl.* 59 67–82. 12698974

[B37] Pecorelli A. Leoni G. Cervellati F. Canali R. Signorini C. Leoncini S. (2013). Genes related to mitochondrial functions, protein degradation, and chromatin folding are differentially expressed in lymphomonocytes of Rett syndrome patients. *Mediators Inflamm.* 2013:137629. 10.1155/2013/137629 24453408 PMC3876710

[B38] Pertea M. Kim D. Pertea G. Leek J. T. Salzberg S. L. (2016). Transcript-level expression analysis of RNA-seq experiments with HISAT. StringTie and Ballgown. *Nat. Protoc.* 11 1650–1667. 10.1038/nprot.2016.095 27560171 PMC5032908

[B39] Politis I. Wang L. Turner J. D. Tsang B. K. (1990). Changes in tissue-type plasminogen activator-like and plasminogen activator inhibitor activities in granulosa and theca layers during ovarian follicle development in the domestic hen. *Biol. Reprod.* 42 747–754. 10.1095/biolreprod42.5.747 2116920

[B40] Qin N. Fan X. C. Zhang Y. Y. Xu X. X. Tyasi T. L. Jing Y. (2015). New insights into implication of the SLIT/ROBO pathway in the prehierarchical follicle development of hen ovary. *Poult. Sci.* 94 2235–2246. 10.3382/ps/pev185 26188027

[B41] Ren J. Sun C. Chen L. Hu J. Huang X. Liu X. (2019). Exploring differentially expressed key genes related to development of follicle by RNA-seq in Peking ducks (AnasPlatyrhynchos). *PLoS One* 14:e0209061. 10.1371/journal.pone.0209061 31237879 PMC6592512

[B42] Richards J. S. Ascoli M. (2018). Endocrine, paracrine, and autocrine signaling pathways that regulate ovulation. *Trends Endocrinol. Metab.* 29 313–325. 10.1016/j.tem.2018.02.012 29602523

[B43] Shiue Y. L. Chen L. R. Chen C. F. Chen Y. L. Ju J. P. Chao C. H. (2006). Identification of transcripts related to high egg production in the chicken hypothalamus and pituitary gland. *Theriogenology* 66 1274–1283. 10.1016/j.theriogenology.2006.03.037 16725186

[B44] Simoni M. Gromoll J. Nieschlag E. (1997). The follicle stimulating hormone receptor: biochemistry, molecular biology, physiology, and pathophysiology. *Endocr. Rev.* 18 739–773. 10.1210/edrv.18.6.0320 9408742

[B45] Son W. Y. Das M. Shalom-Paz E. Holzer H. (2011). Mechanisms of follicle selection and development. *Minerva Ginecol.* 63 89–102. 21508900

[B46] Tao Z. Song W. Zhu C. Xu W. Liu H. Zhang S. (2017). Comparative transcriptomic analysis of high and low egg-producing duck ovaries. *Poult. Sci.* 96 4378–4388. 10.3382/ps/pex229 29053813

[B47] Tuem K. B. Atey T. M. (2017). Neuroactive steroids: receptor interactions and responses. *Front. Neurol.* 8:442. 10.3389/fneur.2017.00442 28894435 PMC5581316

[B48] Wang C. Ma W. (2019). Hypothalamic and pituitary transcriptome profiling using RNA-sequencing in high-yielding and low-yielding laying hens. *Sci. Rep.* 9:10285. 10.1038/s41598-019-46807-3 31311989 PMC6635495

[B49] Wang L. Feng Z. Wang X. Wang X. Zhang X. (2010). DEGseq: an R package for identifying differentially expressed genes from RNA-seq data. *Bioinformatics* 26 136–138. 10.1093/bioinformatics/btp612 19855105

[B50] Woods D. Johnson A. (2005). Regulation of follicle-stimulating hormone-receptor messenger RNA in hen granulosa cells relative to follicle selection. *Biol. Reprod.* 72 643–650. 10.1095/biolreprod.104.033902 15537865

[B51] Wu Y. Zhao X. Chen L. Wang J. Duan Y. Li H. (2020). Transcriptomic analyses of the hypothalamic-pituitary-gonadal axis identify candidate genes related to egg production in Xinjiang Yili geese. *Animals* 10:90. 10.3390/ani10010090 31935822 PMC7023467

[B52] Xu R. F. Qin N. Xu X. X. Sun X. Chen X. Zhao J. (2018). Inhibitory effect of SLIT2 on granulosa cell proliferation mediated by the CDC42-PAKs-ERK1/2 MAPK pathway in the prehierarchical follicles of the chicken ovary. *Sci. Rep.* 8:9168. 10.1038/s41598-018-27601-z 29907785 PMC6003946

[B53] Yu Z. Poppe J. L. Wang X. (2013). Mitochondrial mechanisms of neuroglobin’s neuroprotection. *Oxid Med Cell Longev.* 2013:756989. 10.1155/2013/756989 23634236 PMC3619637

[B54] Zhang T. Chen L. Han K. Zhang X. Zhang G. Dai G. (2019). Transcriptome analysis of ovary in relatively greater and lesser egg producing Jinghai Yellow Chicken. *Anim. Reprod. Sci.* 208:106114. 10.1016/j.anireprosci.2019.106114 31405454

[B55] Zheng Y. Ma L. Liu N. Tang X. Guo S. Zhang B. (2019). Autophagy and apoptosis of porcine ovarian granulosa cells during follicular development. *Animals* 9:1111. 10.3390/ani9121111 31835576 PMC6940823

