# Supplementary figures and images for: Transcriptome Analysis of Ovarian Follicles Reveals Potential Pivotal Genes Associated With Increased and Decreased Rates of Chicken Egg Production
Source: Front Genet. 2021 Mar 10;12:622751. doi: 10.3389/fgene.2021.622751 (PMC7987945; doi:10.3389/fgene.2021.622751)

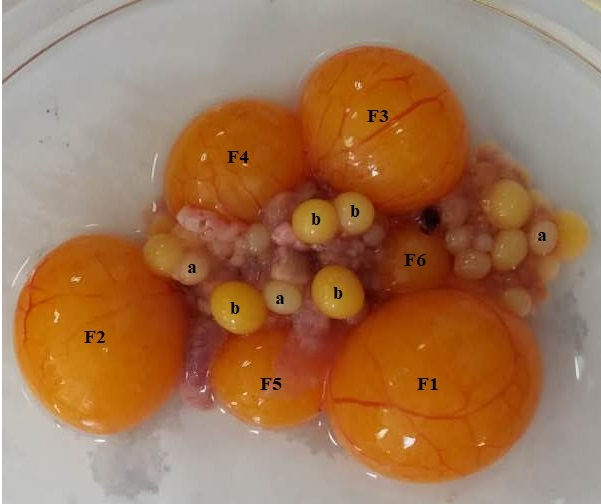

Supplement: Supplementary Figure 1 — Characterization of ovarian follicular morphology in the laying hens sampled. a, White follicles 3.5–5.5 mm in diameter were classified as large white follicles (LWF). b, Yellow follicles 6.0–8.0 mm were grouped as small yellow follicles (SYF). The hierarchical follicles were categorized as F6, F5… and F1 according to their diameters from the smallest to the largest, in which the follicle F6 were collected to yellow follicles 8.5–10.5 mm, and was sampled as large yellow follicles (LYF) in this study. [file Image_1.TIF]

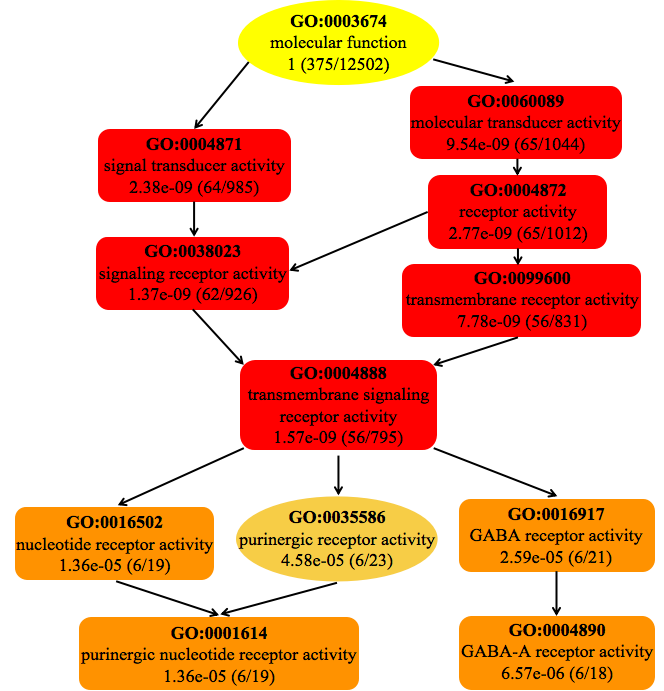

Supplement: Supplementary Figure 2 — The gene ontology DAG structure for hypotaxis of the significant top 10 GO terms of molecular function. [file Image_2.TIF]
