# Supplementary material for: Transcriptome Analysis of Ovarian Follicles Reveals Potential Pivotal Genes Associated With Increased and Decreased Rates of Chicken Egg Production
Source: Front Genet. 2021 Mar 10;12:622751. doi: 10.3389/fgene.2021.622751 (PMC7987945; doi:10.3389/fgene.2021.622751)
Supplement: Supplementary Table 1 — Quality of sequenced raw data. [file Data_Sheet_1.docx]

**TABLE S1** Quality of sequenced raw data.

| Sample | Reads No. | Bases (bp) | Q30 (bp) | N (%) | Q20 (%) | Q30 (%) |
| --- | --- | --- | --- | --- | --- | --- |
| H11 | 43733718 | 6603791418 | 6199293213 | 0.00617 | 97.40 | 93.87 |
| H12 | 41995086 | 6341257986 | 5966922278 | 0.006627 | 97.51 | 94.09 |
| H13 | 47000968 | 7097146168 | 6677773334 | 0.005998 | 97.51 | 94.09 |
| H21 | 45948724 | 6938257324 | 6524867959 | 0.006043 | 97.49 | 94.04 |
| H22 | 42429654 | 6406877754 | 6039118017 | 0.007134 | 97.60 | 94.25 |
| H23 | 42240420 | 6378303420 | 6011899216 | 0.006733 | 97.60 | 94.25 |
| H31 | 52139154 | 7873012254 | 7468415135 | 0.001907 | 97.85 | 94.86 |
| H32 | 44754094 | 6757868194 | 6415316238 | 0.002046 | 97.89 | 94.93 |
| H33 | 41616224 | 6284049824 | 5949809438 | 0.002129 | 97.76 | 94.68 |
| L11 | 53221674 | 8036472774 | 7594588074 | 0.001829 | 97.68 | 94.50 |
| L12 | 48334448 | 7298501648 | 6884241037 | 0.001455 | 97.59 | 94.32 |
| L13 | 46338578 | 6997125278 | 6576885514 | 0.001313 | 97.45 | 93.99 |
| L21 | 44628354 | 6738881454 | 6356877136 | 0.001554 | 97.61 | 94.33 |
| L22 | 48058980 | 7256905980 | 6849495642 | 0.001467 | 97.62 | 94.38 |
| L23 | 48995084 | 7398257684 | 6947945475 | 0.001429 | 97.37 | 93.91 |
| L31 | 41996710 | 6341503210 | 5954135809 | 0.001691 | 97.36 | 93.89 |
| L32 | 43907070 | 6629967570 | 6224459200 | 0.001416 | 97.35 | 93.88 |
| L33 | 45544892 | 6877278692 | 6468856591 | 0.001651 | 97.45 | 94.06 |

Notes: H and L, indicate samples harvested from HR and LR laying hens. First digits, 1, 2 and 3 mark the follicle type of LWF, SYF and LYF. Second digits, 1, 2 and 3 indicate follicles are from different hens.

**TABLE S2** Cleaned data of reads filtered

| Sample | Clean reads No. | Clean data (bp) | Clean reads (%) | Clean data (%) |
| --- | --- | --- | --- | --- |
| H11 | 43457970 | 6542567926 | 99.36 | 99.07 |
| H12 | 41750694 | 6285289046 | 99.41 | 99.11 |
| H13 | 46722392 | 7033912150 | 99.40 | 99.10 |
| H21 | 45674436 | 6875717490 | 99.40 | 99.09 |
| H22 | 42199084 | 6352676798 | 99.45 | 99.15 |
| H23 | 42011124 | 6323848904 | 99.45 | 99.14 |
| H31 | 51902968 | 7812567910 | 99.54 | 99.23 |
| H32 | 44559406 | 6708743716 | 99.56 | 99.27 |
| H33 | 41417292 | 6236982404 | 99.52 | 99.25 |
| L11 | 52955756 | 7973000446 | 99.50 | 99.21 |
| L12 | 48010434 | 7230278376 | 99.32 | 99.06 |
| L13 | 46024668 | 6932185704 | 99.32 | 99.07 |
| L21 | 44326682 | 6676460600 | 99.32 | 99.07 |
| L22 | 47741962 | 7190302714 | 99.34 | 99.08 |
| L23 | 48609428 | 7321313074 | 99.21 | 98.95 |
| L31 | 41657196 | 6274749870 | 99.19 | 98.94 |
| L32 | 43542932 | 6557837122 | 99.17 | 98.91 |
| L33 | 45189634 | 6806288406 | 99.21 | 98.96 |

Notes: H and L, indicate samples harvested from HR and LR laying hens. First digits, 1, 2 and 3 mark the follicle type of LWF, SYF and LYF. Second digits, 1, 2 and 3 indicate follicles are from different hens.

**TABLE S3** Results of the RNASeq map

| Sample | Clean reads | Total mapped | Multiple mapped | Uniquely mapped |
| --- | --- | --- | --- | --- |
| H11 | 43457970 | 36989154 (85.11%) | 1380227 (3.73%) | 35608927 (96.27%) |
| H12 | 41750694 | 35669568 (85.43%) | 1383900 (3.88%) | 34285668 (96.12%) |
| H13 | 46722392 | 40031782 (85.68%) | 1735209 (4.33%) | 38296573 (95.67%) |
| H21 | 45674436 | 39059946 (85.52%) | 1603217 (4.10%) | 37456729 (95.90%) |
| H22 | 42199084 | 36169975 (85.71%) | 1537030 (4.25%) | 34632945 (95.75%) |
| H23 | 42011124 | 35837904 (85.31%) | 1813275 (5.06%) | 34024629 (94.94%) |
| H31 | 51902968 | 44549945 (85.83%) | 1689968 (3.79%) | 42859977 (96.21%) |
| H32 | 44559406 | 38209439 (85.75%) | 1608308 (4.21%) | 36601131 (95.79%) |
| H33 | 41417292 | 35483457 (85.67%) | 1861444 (5.25%) | 33622013 (94.75%) |
| L11 | 52955756 | 45212064 (85.38%) | 1808582 (4.00%) | 43403482 (96.00%) |
| L12 | 48010434 | 41237426 (85.89%) | 1752663 (4.25%) | 39484763 (95.75%) |
| L13 | 46024668 | 39390948 (85.59%) | 1817557 (4.61%) | 37573391 (95.39%) |
| L21 | 44326682 | 38338558 (86.49%) | 1443183 (3.76%) | 36895375 (96.24%) |
| L22 | 47741962 | 40955171 (85.78%) | 1448845 (3.54%) | 39506326 (96.46%) |
| L23 | 48609428 | 41443727 (85.26%) | 1783090 (4.30%) | 39660637 (95.70%) |
| L31 | 41657196 | 35554659 (85.35%) | 1430900 (4.02%) | 34123759 (95.98%) |
| L32 | 43542932 | 37123950 (85.26%) | 1633596 (4.40%) | 35490354 (95.60%) |
| L33 | 45189634 | 38683810 (85.60%) | 1831347 (4.73%) | 36852463 (95.27%) |

Notes: H and L, indicate samples harvested from HR and LR laying hens. First digits, 1, 2 and 3 mark the follicle type of LWF, SYF and LYF. Second digits, 1, 2 and 3 indicate follicles are from different hens.

**TABLE S4** Results of the RNASeq mapped events

| Sample | Map events | Mapped to gene | Mapped to InterGene | Mapped to exon |
| --- | --- | --- | --- | --- |
| H11 | 35608927 | 29369862 (82.48%) | 6239065 (17.52%) | 26510359 (90.26%) |
| H12 | 34285668 | 28340142 (82.66%) | 5945526 (17.34%) | 25409832 (89.66%) |
| H13 | 38296573 | 31723115 (82.84%) | 6573458 (17.16%) | 28630012 (90.25%) |
| H21 | 37456729 | 30952879 (82.64%) | 6503850 (17.36%) | 27829281 (89.91%) |
| H22 | 34632945 | 28517481 (82.34%) | 6115464 (17.66%) | 25263573 (88.59%) |
| H23 | 34024629 | 28108436 (82.61%) | 5916193 (17.39%) | 25039287 (89.08%) |
| H31 | 42859977 | 35567023 (82.98%) | 7292954 (17.02%) | 32496583 (91.37%) |
| H32 | 36601131 | 30001784 (81.97%) | 6599347 (18.03%) | 26267281 (87.55%) |
| H33 | 33622013 | 27467363 (81.69%) | 6154650 (18.31%) | 24797175 (90.28%) |
| L11 | 43403482 | 35621532 (82.07%) | 7781950 (17.93%) | 32247480 (90.53%) |
| L12 | 39484763 | 32479244 (82.26%) | 7005519 (17.74%) | 28782384 (88.62%) |
| L13 | 37573391 | 31023443 (82.57%) | 6549948 (17.43%) | 27919716 (90.00%) |
| L21 | 36895375 | 30731721 (83.29%) | 6163654 (16.71%) | 27764796 (90.35%) |
| L22 | 39506326 | 32457664 (82.16%) | 7048662 (17.84%) | 29169999 (89.87%) |
| L23 | 39660637 | 32577657 (82.14%) | 7082980 (17.86%) | 29072475 (89.24%) |
| L31 | 34123759 | 27909689 (81.79%) | 6214070 (18.21%) | 24340522 (87.21%) |
| L32 | 35490354 | 29096527 (81.98%) | 6393827 (18.02%) | 25576872 (87.90%) |
| L33 | 36852463 | 30503076 (82.77%) | 6349387 (17.23%) | 27273822 (89.41%) |

Notes: H and L, indicate samples harvested from HR and LR laying hens. First digits, 1, 2 and 3 mark the follicle type of LWF, SYF and LYF. Second digits, 1, 2 and 3 indicate follicles are from different hens.
