# Supplementary material for: Transcriptome Analysis of Ovarian Follicles Reveals Potential Pivotal Genes Associated With Increased and Decreased Rates of Chicken Egg Production
Source: Front Genet. 2021 Mar 10;12:622751. doi: 10.3389/fgene.2021.622751 (PMC7987945; doi:10.3389/fgene.2021.622751)
Supplement: Supplementary Table 11 — Enriched KEGG pathways and DEGs in ovarian LWF follicles of HR and LR hens. [file Data_Sheet_2.docx]

**TABLES11**Enriched KEGG pathways and DEGs in ovarian LWF folliclesof HR and LR hens.

| PathwayID | Pathway | DEG  No. | UpGene | Down_Gene | Up_KO | Down_KO |
| --- | --- | --- | --- | --- | --- | --- |
| ko04514 | Cell adhesion molecules | 6 | DMB2, ICOS,LRRC4C | CDKN1A, CREB3L3,RBPJL | K07523,K06713,K06752 | K06747,K06491,K06752 |
| ko03320 | PPAR signaling pathway | 4 | ACSBG1 | ACSBG2,SCD,RXRG | K15013 | K08526,K00507,K15013 |
| ko04080 | Neuroactive ligand-receptor interaction | 9 | GRIK1,GABRA1,VIPR2, GALR2 | MC2R,OPRD1,F2RL1,P2RX1,CHRNA4 | K05175,K04590,K05201,K04231 | K05215,K04200,K04806,K04234,K04213 |
| ko00061 | Fatty acid biosynthesis | 2 | ACSBG1 | ACSBG2 | K15013 | K15013 |
| ko04152 | AMPK signaling pathway | 5 | CAB39L,PIK3R1,FBP1, | CREB3L3,SCD | K08272,K02649,K03841 | K00507,K09048 |
| ko04024 | cAMP signaling pathway | 6 | RYR2, VIPR2,PIK3R1, | MC2R,CREB3L3 | K04590,K05852,K02649,K04962 | K09048,K04200 |
| ko04920 | Adipocytokine signaling pathway | 3 | ACSBG1 | ACSBG2,RXRG, | K15013 | K08526,K15013 |
| ko04512 | ECM-receptor interaction | 3 | COL9A2, COL2A1,VTN | ─ | K19719,K08131,K06251 | ─ |
| ko04115 | p53 signaling pathway | 3 | PERP1,TP53I3 | CDKN1A | K10136,K10133 | K06625 |
| ko04911 | Insulin secretion | 3 | GCG,RYR2 | CREB3L3 | K05259,K04962 | K09048 |

**TABLE S12**Enriched KEGG pathways and DEGs in ovarian SYF folliclesof HR and LR hens.

| PathwayID | Pathway | DEG  No. | UpGene | Down_Gene | Up_KO | Down_KO |
| --- | --- | --- | --- | --- | --- | --- |
| ko04145 | phagosome | 20 | MARCO, MMR1L3, TAP1,C1R, TLR2A,NCF4,CYBB,ITGB2,ENSGALG00000029381,BLB1,ENSGALG00000030368, BF1,TLR4,CD36,CTSS, BF2,MMR1L4,MMR1L,ENSGALG00000028357 | TUBA1C | K01330,K10160,K21421,K06560,K06751,K06259,K05654,K10159,K05653,K01368,K08012,K06464,K13884,K06752 | K07374 |
| ko04514 | Cell adhesion molecules | 13 | ENSGALG00000030368, BLB1,BF1,NRCAM,VCAM1, PTPRC,CNTN1,BF2, CD86, CLDN2,ALCAM,ITGB2 | NCAM2 | K05413,K06756,K06751,K06547,K06087,K06478,K06464,K06527,K06752,K06759 | K06491 |
| ko04064 | NF-kappa B signaling pathway | 10 | BTK,TRAF5,TLR4,VCAM1,BCL2A1,LYN, ZAP70, LY96, CCL4, SYK | _ | K05854,K10160,K07370,K05855,K02162,K09849,K12964,K05400,K07360,K06527 | _ |
| ko04020 | Calcium signaling pathway | 13 | BDKRB1,PTK2B, DRD1,HTR2A,PTAFR,ADCY7,SLC8A1,HRH1, HRH2,P2RX1,P2RX7 | GRPR, CCKBR | K08047,K04279,K04150,K03915,K04149,K05215,K05871,K04157,K05849,K04144,K05220 | K04169,K04195 |
| ko04620 | Toll-like receptor signaling pathway | 8 | IRF7,TLR4,TLR2A,CD86,LY96,TLR7,CCL4,IKBKE | _ | K05413,K09447,K10160,K10159,K12964,K05400,K07211,K05404 | _ |
| ko04080 | Neuroactive ligand-receptor interaction | 17 | BDKRB1,LOC427545,F2RL2,P2RY13,GABRA1, DRD1, PTAFR,HTR2A,GABRB2,HRH1,HRH2,GABRG1, P2RX1,GABRQ,P2RX7 | CCKBR,GRPR | K04279,K05186,K04150,K05215,K03915,K04149,K08388,K04151,K05192,K05175,K04157,K04235,K04144,K05181,K05220 | K04169,K04195 |
| ko04727 | GABAergic synapse | 6 | GABRA1,GABRG1,GABRB2,ADCY7,GABRQ | GNG13 | K05175,K08047,K05186,K05192,K05181 | K04547 |

**TABLES13**Enriched KEGG pathways and DEGs in ovarian LYF folliclesof HR and LR hens.

| PathwayID | Pathway | DEG  No. | UpGene | Down_Gene | Up_KO | Down_KO |
| --- | --- | --- | --- | --- | --- | --- |
| ko04080 | neuroactive ligand-receptor interaction | 21 | TSHB, GRIA2, GHRHR-LR, VIP, GABRB2, HTR1A,GRIN2A,LEPR, PRLR, GABRA1,TRH, AGTR2, GRIA4,HRH1,NPY4R, CHRM5,CCKAR,GRM4 | GABRD, SSTR2, CCKBR | K04133,K04149,K04153,K04167,K04589,K04194,K05251,K05062,K05181,K05200,K05253,K05264,K05081,K04206,K05209,K05175,K05198,K04607 | K04218,  K05184,  K04195 |
| ko04514 | CAMs | 7 | NLGN1,VCAN,NCAM1,CNTNAP2 | ICOS, NRXN1, NCAM2 | K06793,K07378,K06752,K07380 | K06491,  K06713,  K07377 |
| ko04713 | circadian entrainment | 6 | GRIA2, RASD1,GRIN2A, RYR2, GUCY1A2,GRIA4 | _ | K05198,K12318,K05200,K07843,K05209,K04962 | _ |
| ko04724 | glutamatergic synapse | 6 | GRIA2, GRIN2A, SLC1A1, SLC1A2, GRM4,GRIA4 | ­_ | K05612,K05198,K05200,K05613,K05209,K04607 | ­_ |
| ko00512 | mucin type O-glycan biosynthesis | 3 | GALNT3, GCNT4 | ST6GALNAC1 | K09663,K00710 | K03479 |
| ko03320 | PPAR signaling pathway | 4 | FABP7,ACSBG1 | ACOX2,FABP3 | K08756,K15013 | K10214,  K08752 |
| ko00472 | D-Arginine and D-ornithine metabolism | 1 | _ | DAO | _ | K00273 |
| ko04973 | carbohydrate digestion and absorption | 3 | LCT, PIK3R1 | SLC5A1 | K01229,K02649 | K14158 |
| ko04630 | JAK-STAT signaling pathway | 6 | CNTFR,LEPR, IL7R,PIK3R1, PRLR | CDKN1A | K05072,K05081,K02649,K05059,K05062 | K06625 |
| ko04024 | cAMP signaling pathway | 7 | GRIA2,GRIA4,HTR1A, GRIN2A, RYR2, PIK3R1 | SSTR2 | K05198,K05200,K04153,K02649,K05209,K04962 | K04218 |
| ko04060 | cytokine-cytokine receptor interaction | 7 | BMPR2, CNTFR, IL16, LEPR, IL7R, PRLR | EDA2R | K22628,K05072,K05081,K05059,K05062,K04671 | K05163 |
